# Comparative Analysis of Public RNA-Sequencing Data from Human Intestinal Enteroid (HIEs) Infected with Enteric RNA Viruses Identifies Universal and Virus-Specific Epithelial Responses

**DOI:** 10.3390/v13061059

**Published:** 2021-06-03

**Authors:** Roberto J. Cieza, Jonathan L. Golob, Justin A. Colacino, Christiane E. Wobus

**Affiliations:** 1 Department of Microbiology and Immunology, University of Michigan, Ann Arbor, MI 48109, USA; rjcieza@med.umich.edu; 2 Department of Internal Medicine, Division of Infectious Diseases, University of Michigan Medical School, Ann Arbor, MI 48109, USA; golobj@med.umich.edu; 3 Department of Environmental Health Sciences, University of Michigan, Ann Arbor, MI 48109, USA; colacino@umich.edu; 4 Department of Nutritional Sciences, University of Michigan, Ann Arbor, MI 48109, USA

**Keywords:** astrovirus, norovirus, rotavirus, human intestinal enteroids, transcriptomics, meta-analysis

## Abstract

Acute gastroenteritis (AGE) has a significant disease burden on society. Noroviruses, rotaviruses, and astroviruses are important viral causes of AGE but are relatively understudied enteric pathogens. Recent developments in novel biomimetic human models of enteric disease are opening new possibilities for studying human-specific host–microbe interactions. Human intestinal enteroids (HIE), which are epithelium-only intestinal organoids derived from stem cells isolated from human intestinal biopsy tissues, have been successfully used to culture representative norovirus, rotavirus, and astrovirus strains. Previous studies investigated host–virus interactions at the intestinal epithelial interface by individually profiling the epithelial transcriptional response to a member of each virus family by RNA sequencing (RNA-seq). Despite differences in the tissue origin, enteric virus used, and hours post infection at which RNA was collected in each data set, the uniform analysis of publicly available datasets identified a conserved epithelial response to virus infection focused around “type I interferon production” and interferon-stimulated genes. Additionally, transcriptional changes specific to only one or two of the enteric viruses were also identified. This study can guide future explorations into common and unique aspects of the host response to virus infections in the human intestinal epithelium and demonstrates the promise of comparative RNA-seq analysis, even if performed under different experimental conditions, to discover universal and virus-specific genes and pathways responsible for antiviral host defense.

## 1. Introduction

Acute gastroenteritis (AGE) is a major source of illness globally. It is defined as the inflammation of the mucus membranes of the intestinal tract accompanied by rapid onset diarrhea, nausea, vomiting, and abdominal pain. Five major virus families have been identified as etiological agents of viral gastroenteritis: noroviruses, sapoviruses (both single-stranded positive-sense RNA viruses belonging to the *Caliciviridae* family), rotaviruses (double-stranded RNA viruses belonging to the *Reoviridae* family), adenoviruses (double-stranded DNA viruses belonging to the *Adenoviridae* family), and astroviruses (single-stranded RNA viruses belonging to the family *Astroviridae*) [1]. Only 20–30% of AGE cases in the United States have a specific causal virus identified [2]. Globally, noroviruses have the highest prevalence across all age groups, causing approximately one fifth of all AGE cases [3]. Rotaviruses are the leading cause of AGE in children < 5 years of age despite the availability of a vaccine [4]. Human astroviruses typically cause diarrhea in children < 2 years of age but extraintestinal disease, including meningitis/encephalitis, is observed in immunocompromised individuals [5]. A full understanding of viral AGE intestinal pathogenesis has been challenging due to genetic diversity within each viral family and due to the lack of suitable experimental models resembling the complexity of the human intestinal epithelium.

Human intestinal enteroids (HIEs) are patient-derived endoderm-only 3D structures composed of heterogeneous cell populations recapitulating intestinal tissues in vivo, providing a more faithful experimental model than immortalized and transformed cells. A large degree of epithelial cellular diversity is observed in HIEs: Columnar intestinal epithelial, stem/progenitor, enteroendocrine, and secretory cells can be observed depending on the culture conditions [6]. Spheroid structures can also be transitioned into 2D monolayers to facilitate host–microbe interaction studies in physiologic asymmetric oxygen conditions [7]. Furthermore, HIEs have been used to study the pathogenesis of several enteric viruses including difficult-to-cultivate ones [8,9].

HIEs provide a uniquely complex system to study host–virus interactions at the intestinal epithelial interface and have been used to profile the epithelial transcriptional response to viral causes of AGE by RNA sequencing (RNA-seq) in several studies. Non-classic human astrovirus (VA1 strain) upon infection of duodenal HIEs triggered type I and type III interferon (IFN) signaling [10]. Ileal HIEs infected with human norovirus (GII.4 strain) showed high enrichment in STAT1 and STAT2 binding sites, suggesting JAK-STAT signaling pathway activation due to type I IFN signaling [11]. Jejunal HIEs when infected with rotavirus (strain Ito) showed a predominant and conserved type III IFN response [12]. Integrating the conclusions from these studies that explore the pathogenesis of astrovirus [10], norovirus [11], and rotavirus [12] in HIEs is challenging since there are technical differences between studies. Each study uses HIEs grown from different tissue donors and varies the time interval at which the host response to the virus was evaluated. Furthermore, different library construction protocols can result in different sequence coverage, complexity, and evenness [13]. Despite the limitations to the comparative analysis of RNA-seq datasets generated from different studies, a re-analysis of public datasets can provide novel insights into a scientific question as long as researchers take the biological context into consideration in which each dataset was generated [14].

Here, our goal was to re-analyze three publicly available datasets from HIEs infected with three representative viruses that cause AGE to gain further insights into shared and unique transcriptional changes in the intestinal epithelium upon virus infection from a qualitative assessment standpoint.

## 2. Materials and Methods

### 2.1. Cell Lines and Virus Infection

Each public RNA-seq dataset was generated in a different institution with variations in the experimental protocol used for viral infection, which we briefly summarize here. To generate the RNA-seq dataset from duodenal HIEs infected with astrovirus, HIEs were seeded in 48-well plates as 2D monolayers in complete L-WRN medium (GMCF+) and infected with astrovirus strain VA1 at an MOI of 1 based on genome copies per cell for 1 h at 37 °C or mock. Total RNA was extracted for sequencing at 24 h post-infection (hpi) [10]. Ileal HIE monolayers grown in 48-well plates were incubated for 2 h at 37 °C with stool filtrates containing a patient-derived GII.4 human norovirus strain (~1 × 10^6^ viral RNA copies) or mock-treated. Samples were collected at 48 hpi before sequencing [11]. For the RNA-seq dataset generated from rotavirus-infected jejunal HIEs, spheroid (3D) HIEs were grown in complete media with growth factors (CMGF+) and differentiated for 3–4 days before infection. Then HIEs were infected with human rotavirus (HRV) strain Ito and sequenced at 6 hpi [12].

### 2.2. Selection of GEO and EMBL-EBI Data

The sequencing reads obtained from RNA-seq experiments in each one of the studies re-analyzed were obtained from the Gene Expression Omnibus (GEO) or the European Bioinformatics Institute (EMBL-EBI) ArrayExpress collection. FASTQ files from a total of 12 HIEs samples (6 mock-treaded and 6 virus-infected) grown from two patients (TI006 and TI365) were obtained for the norovirus-infected ileal HIEs study from the GEO database (accession number GSE117911) [11]. FASTQ files for the rotavirus-infected jejunal HIEs study (accession number GSE90796) were also obtained from the GEO database. A total of 4 HIEs samples (2 mock and 2 treated) obtained from HIEs grown from two patients (J2 and J11) were deposited under this accession number [12]. Finally, for the astrovirus-infected duodenal HIE study, FASTQ files were obtained from EMBL-EBI ArrayExpress collection for a total of 6 HIE samples (3 mock-treated and 3 virus-infected) grown from one patient (D124) [10]. The metadata associated with each one of the samples used in this study is listed in Supplemental Table S1.

### 2.3. Differential Expression Analysis

Transcript abundances from pseudoalignments (provided in transcripts-per-million) were generated for each RNA-seq dataset using Kallisto [15] and used to generate matrices with estimated gene counts for each one of the RNA-seq datasets re-analyzed. We took into account whether the publicly available RNA-seq data was generated from paired-end or single-end Illumina sequencing.

Estimated gene count matrices were then generated for each RNA-seq dataset from transcript abundances using the package tximport [16]. Subsequently, we independently filtered out weakly expressed genes by calculating a similarity index among biological replicates using the HTSFilter method which seeks to identify whether genes tend to either have normalized counts less than or equal to the cutoff value in all samples (filtered genes) or greater than the cutoff value in all samples (non-filtered genes) [17]. The set of genes used for differential expression analysis for each RNA-seq dataset consisted then of non-weakly expressed genes that had an annotation in the Entrez Molecular Sequence Database System [18] and a symbol in HUGO Gene Nomenclature Committee (HGNC) [19].

Filtered estimated gene count matrices generated for each RNA-seq dataset were normalized (median of ratios) with the R package DESeq2 version 1.32.0 (https://bioconductor.org/packages/release/bioc/vignettes/DESeq2/inst/doc/DESeq2.html) [20] and subjected to differential expression analysis using the alternative shrinkage estimator ashr [21] to control for false discovery rates (FDR), and effect sizes. A cutoff of an actual fold-change of at least 1.5 (Log_2_ FC > 0.58) with a false discovery rate (FDR) cutoff of 1% (adjusted *p*-value < 0.01) was used to determine whether a gene was differentially expressed. Volcano plots were generated to display the list of DEG for each RNA-seq dataset using the R package EnhancedVolcano [22]. Additionally, we evaluated the association of DEGs with type I, II, III IFN responses using the Interferome database (http://www.interferome.org/interferome/home.jspx last accessed on 30 March 2021).

Estimated gene count matrices were also used for the exploratory analysis of the dimensionality of the data [23]. Dimensionality reduction was done via two techniques: Principal Component Analysis (PCA) and T-Distributed Stochastic Neighbouring Entities (t-SNE). Each RNA-seq dataset was explored individually to determine the variance within each dataset due to tissue origin of HIEs and treatment modality (mock-treatment versus viral infection). PCA plots and t-SNE plots were generated using the R packages factoextra and Rtsne available at https://rpkgs.datanovia.com/factoextra/index.html and https://github.com/jkrijthe/Rtsne accessed on 30 March 2021, respectively.

### 2.4. Over-Representation Analysis

Over-representation analysis was used to determine whether known biological processes (BP) were enriched in a list of differentially expressed genes (DEGs) for each one of the RNA-seq datasets analyzed. The package clusterProfiler [24] was used for over-representation analysis with strict false discovery rate (FDR) adjusted *p*-value cutoff of <0.001 using the Benjamini–Hochberg (BH) step-up procedure. To determine whether any BP categories were enriched, the gene ratio (number of DEGs relative to gene ontology [GO term]/total number of DEGs) and background ratio (number of genes relative to GO term/total number of DEGs and non-DEGs) are calculated and then compared. Visualization of the DEG assigned to each BP category was done using the R package ComplexHeatmap version 2.6.2 [25] available at https://github.com/jokergoo/ComplexHeatmap.

### 2.5. Transcription Factor Enrichment Analysis (TFEA)

TFEA using the R package TFEA.ChIP [26] was used to identify transcription factors (TF) responsible fo the co-regulation of differentially expressed genes (DEGs) shared across RNA-seq datasets as well as for DEGs identified uniquely in a single dataset. TFEA.ChIP predicts transcription factor binding sites (TFBS) proximal to a given set of genes (DEGs) based on experimentally determined protein-DNA binding profiles from chromatin immunoprecipitation (ChIP) sequencing (ChIP-seq). A total of 1122 ChIP-seq datasets covering 333 different TFs generated by the ENCODE Consortium [27], as well as collected from the Gene Expression Omnibus (GEO), comprise the TFBS database used by TFEA.ChIP. The analysis of the association of TFBS consisted of comparing how many targets of a given TF are in two lists of genes of interest (DEGs versus non-DEGs). A contingency matrix was generated for each ChIP-seq in the TFBS database counting the number of genes it interacts with in the DEGs list and non-DEGs list, with a subsequent Fisher’s exact test applied to each contingency matrix to determine if the difference in the distribution is statistically significant. The results were subsequently summarized by TF. Gene Set Enrichment Analysis (GSEA) is then performed to test whether ChIP-seq datasets belonging to the same TF are significantly enriched or depleted as a group. Enrichment Score (ES) values reflect the degree to which a TF is over-represented at the top (up-regulated) or bottom (down-regulated) of a ranked list. The enriched TF ranking as well as the number of ChIP-seq datasets contributing to the TF enrichment were subsequently graphed with ggplot2 in R.

### 2.6. Code Availability

The source code used for analysis of the datasets and to generate the figures in this study are available at: https://gitlab.com/rjcieza/HIEs-GI-viruses-comparative-transcriptomic.git, accessed on 30 March 2021. The datasets that have been used in this study are publicly available from the GEO and EMBL-EBI as indicated above.

## 3. Results

### 3.1. Samples Across Datasets Showed Divergence Due to Tissue Origin of the HIEs Line and Viral Infection

**Figure 1:**
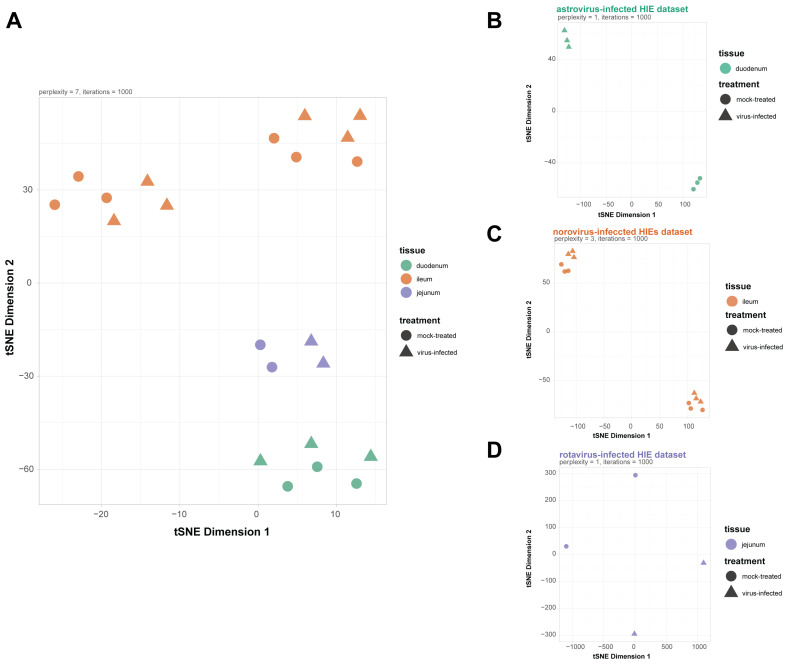
Samples across the three datasets showed divergence due to tissue origin of the HIE line but due to viral infection when looking at each dataset individually. Nonlinear dimensionality reduction with t-SNE of the 16 samples belonging to the three datasets were analyzed. Samples are colored by the segment of the small intestine that was used to derive HIE and each one of the datasets corresponds to a different small intestinal segment (duodenum, ileum and jejunum). The t-SNE plot shows that clustering is primarily driven by the tissue origin of HIE (**A**). Nonlinear dimensionality reduction with t-SNE for each individual dataset (6, 12 and 4 samples for astrovirus-infected (**B**), norovirus-infected (**C**), and rotavirus-infected (**D**) HIEs datasets, respectively) shows clustering of samples due to treatment (mock-treated versus virus-infected) as well as the HIE line since in the norovirus-infected and rotavirus-infected HIE datasets more than one HIE line were used. Plots were generated with Rtsne and perplexity values were chosen for each plot after stability.

The RNA-seq datasets from each of the studies that we analyzed [10,11,12] were generated from HIEs grown from small intestinal biopsy tissue. However, in each study, a different section of the small intestine from a different donor was used, specifically the terminal ileum (TI006 and TI365 lines) for norovirus infection [11], jejunum (J2 and J11 lines) for rotavirus infection [12], and duodenum (D124) for astrovirus infection [10] (Supplemental Table S1). Hence, it was not surprising that the main source of variance when looking at the three datasets together was not the treatment (mock-treated versus virus-infected) but rather the segment of the small intestine used to derive HIEs (Figure 1A). In the datasets where more than one HIE line was used, the driver of variance still was the HIE line used in the experiment (although that is linked with the institution where the RNA-seq experiment was done). Nevertheless, when looking at each dataset individually and comparing between samples infected or not, consistent differences can be observed (Figure 1B–D). Dimensional reduction via principal component analysis (PCA) confirmed these results and validated that the primary source of variance in the three datasets was the tissue origin of the small intestine used to derive HIEs (Supplemental Figure S1).

### 3.2. Most Non-Weakly Expressed Genes are Shared Across Datasets Including a Number of Innate Immune Response Genes

**Figure 2:**
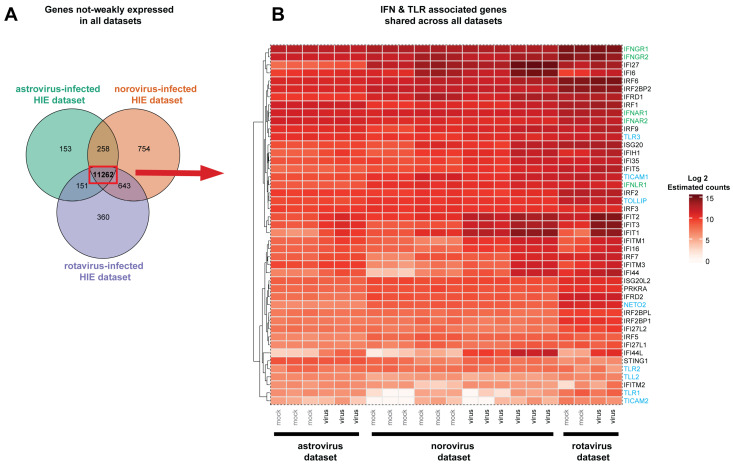
A large degree of overlap of non-weakly expressed genes is observed across all datasets. Estimated gene count matrices for each RNA-seq dataset were filtered to remove genes with low constant expression levels (non-weakly expressed) with the package HTSFilter in R. Log transformed estimated counts for selected genes associated with IFN and Toll-like receptor signaling are shown. (**A**) A total of 11,262 shared genes were identified in the three RNA-seq datasets, and the norovirus-infected ileal HIEs contained the largest number of genes identified in a single dataset. (**B**) Genes detected in all three datasets and annotated in the Entrez Molecular Sequence Database System as part of the IFN response or involved in Toll-like receptor signaling are shown. Each column shows individual replicates (mock-treated samples in grey and virus-infected samples in black) for each study. Highlighted in green are genes coding for IFN or IFN receptors, while genes associated with Toll-like receptor signaling are in blue.

We next focused on identifying differences and similarities in HIE transcriptional changes upon enteric viral infection between the three RNA-seq datasets analyzed. After filtering we considered 11,824, 12,917, and 12,416 genes from astrovirus-infected duodenal HIEs, norovirus-infected ileal HIEs and rotavirus-infected jejunal HIEs, respectively. The union for the three RNA-seq datasets consisted of 13,581 genes with 82.92% of these (11,262 genes) present in all the datasets (Figure 2A). Among the non-weakly expressed genes detected in all RNA-seq datasets were several IFN receptor genes (IFNAR, IFNGR, and IFNLR) as well as IFN-stimulated genes. Other key players of the innate immune response, such as the genes associated with Toll-like receptor signaling (TLR1-3) (Figure 2B) and chemokines secreted in response to IFN, including CXCL10 and CXCL1 (data not shown), were also detected in all the datasets. Several of the selected shared genes annotated in the Entrez Molecular Sequence Database System as part of IFN responses showed estimated gene counts above 1000 (Log_2_ values > 10). No genes coding for IFNs were found in the list of shared genes from all RNA-seq datasets. However, IFN-λ (IFNL1-3) was uniquely identified in rotavirus-infected jejunal HIEs, while IFN-ε (IFNE) was partially shared across two of the RNA-seq datasets (Supplemental Table S2). Biologically, IFNs clearly play a critical role in limiting astrovirus [10] and norovirus infections [28], but limitations in sensitivity due to sequencing depth of RNA sequencing [29], may account for this discrepancy in detection. Taken together, the overall expression patterns across the different RNA-seq datasets identified a large number of shared genes across the three studies.

### 3.3. Differential Expression Analysis Showed That HIE Responses Against Astrovirus, Norovirus and Rotavirus Is Primarily Characterized by an Up-Regulation of Interferon-Stimulated Genes (ISGs)

To determine the differentially expressed genes (DEGs) between infected versus mock controls in each RNA-seq dataset, we re-analyzed each dataset individually for differential expression with the package DeSeq2 and compared the list of DEGs we obtained in this analysis with those previously published [10,11,12]. For each individual RNA-seq dataset, DEGs were identified among non-weakly expressed genes (Table 1) based on the criteria of an actual fold-change of at least 1.5 (Log_2_ FC > 0.58) with a false discovery rate (FDR) cutoff of 1% (adjusted *p*-value < 0.01) using an Empirical Bayes (EB) approach with the R package ashr [21]. Relatively stringent FDR cutoffs were used to focus only on the strongest biological effects upon virus infection in each of the datasets.

**Figure 3:**
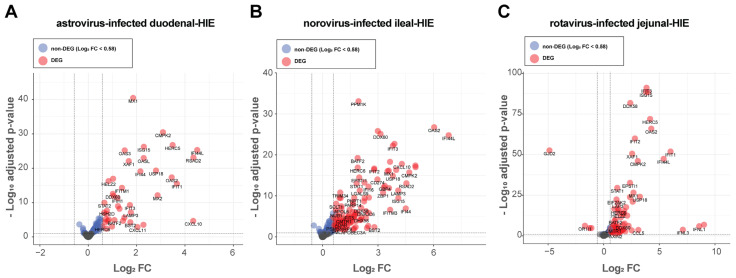
Differential expression analysis in HIEs infected with astrovirus, norovirus and rotavirus. Differentially expressed genes (DEGs) for the three re-analyzed RNA-seq datasets are shown. DEGs with Log_2_ fold-change (FC) > 0.58 when compared to mock-treated samples and an FDR of less than 1% are shown red, while genes with an FDR of less than 1% (adjusted *p*-value < 0.01) that were below the Log_2_ FC threshold are shown in blue. FDR was calculated using an Empirical Bayes (EB) approach with the R package ashr. DEGs were identified in astrovirus-infected duodenal-HIEs (**A**), norovirus-infected ileal-HIEs (**B**) and rotavirus-infected jejunal-HIEs (**C**) using the package DESeq2 and plotted using the package EnhanceVolcano in R.

In the astrovirus-infected duodenal HIE dataset, 42 DEGs were identified out of a total of 11,824 non-weakly expressed genes, the smallest number of DEGs of the three RNA-seq datasets analyzed and all were up-regulated genes (Figure 3A). When re-analyzing the norovirus-infected ileal-HIEs dataset, we evaluated the sequencing data from both HIE lines (TI006 and TI365) together to explore universal patterns of host-response against norovirus GII.4. Additionally, sequencing data for both HIE lines were generated within the same institution for the same study and both HIE lines were grown from ileal biopsies. We found that of a total of 12,917 non-weakly expressed genes, 109 were DEGs, which were all up-regulated (Figure 3B). Finally, in the RNA-seq dataset generated from rotavirus-infected jejunal HIEs, two jejunal HIE lines (J2 and J11) were used. In this study, out of a total of 12,416 non-weakly expressed genes, 75 were DEGs with 71 up-regulated and 4 down-regulated genes (Figure 3C).

A large degree of similarity was found in the list of DEGs identified between our analysis and those previously published [10,11,12]. Any differences in the number of DEGs detected between our and published analysis are likely due to different filtering methods applied to the matrices containing the estimated gene counts and different Log_2_ FC cutoff.

### 3.4. Antiviral Defense and IFN Signaling Represent a Conserved Response of the Epithelium to Viral Infection

**Figure 4:**
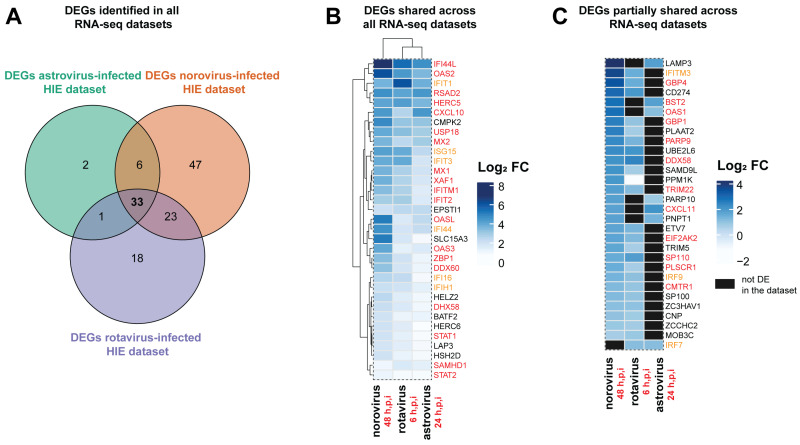
Shared differentially expressed genes (DEGs) across datasets, which are part of the conserved epithelial response to viral infection, are enriched in IFN-associated genes. Shared and unique DEGs across all datasets were determined using the package DESeq2 and heatmaps generated with the package ComplexHeatmap in R. For a gene to be classified as differentially expressed it had to meet the criteria of an actual fold-change of at least 1.5 (Log_2_ FC > 0.58) with a false discovery rate (FDR) cutoff of 1% (adjusted *p*-value < 0.01) using an Empirical Bayes (EB) approach with the R package ashr. The intersection and union of DEGs across the three RNA-seq datasets evaluated is shown (**A**). Expression pattern for DEGs shared across all datasets is shown, with genes annotated in the Entrez Molecular Sequence Database System as part of IFN responses highlighted in red and genes with a symbol containing IFI (IFN-inducible) or ISG (IFN-stimulated genes) in orange (**B**)**.** Partially shared DEGs in two out of three RNA-seq datasets are also shown (**C**). The sampling time point in hours post-infection (h.p.i) at which each RNA-seq dataset was generated is indicated.

To identify shared DEGs, we compared the list of DEGs for each individual RNA-seq dataset. There were 33 DEGs common to all three datasets, suggesting that they are part of a shared response against viruses by the intestinal epithelium (Figure 4A). Twenty-five out of the 33 common DEGs are annotated as part of the IFN response in the Entrez Molecular Sequence Database system (Figure 4B). Of these, a number of IFN-stimulated genes (ISGs) were identified as part of the universal HIEs response to the three viruses, including IFN-induced protein with Tetratricopeptide repeats (*IFIT1*, *IFIT2* and *IFIT3*) as well as the signal transducer and activator of transcription proteins 1 and 2 (*STAT1* and *STAT2*). Shared DEGs were also evaluated in the Interferome database, which revealed that 16 out of the 33 DEGs shared across RNA-seq datasets were associated with type I, II, III IFN responses (Table 2). Hence, the set of shared DEGs across the three datasets was enriched in genes associated with IFN signaling. However, within the shared DEGs, virus-specific induction patterns were also observed. For example, we observed that the ISGs 2′-5′-oligoadenylate synthetase-like (*OASL*) and Interferon Induced Protein 44 (*IFI44*), as well as the Solute Carrier Family 15 Member 3 (*SLC15A3*), which potentiates MAVS- and STING-mediated IFN production [30], showed a stronger up-regulation in the norovirus-infected ileal HIEs compared to astrovirus- and rotavirus-infected ones (Figure 4B). On the other hand, *IFIT1* showed greater up-regulation in the rotavirus dataset versus either the norovirus or astrovirus datasets. In addition, 30 genes were differentially expressed in only two out of the three datasets (Figure 4C). These partially shared DEGs included for example C-X-C Motif Chemokine Ligand 11 (*CXCL11*) and Bone Marrow Stromal Cell Antigen 2 (*BST2*, also called Tetherin), which was up-regulated in response to norovirus and astrovirus but not rotavirus infection. A different pattern was observed for example for the transcription factors IFN Regulated Factor 7 and 9 (*IRF7*, *IRF9*), which were differentially expressed in the astrovirus and rotavirus but not norovirus datasets, and the rotavirus and norovirus but not astrovirus datasets, respectively.

**Figure 5:**
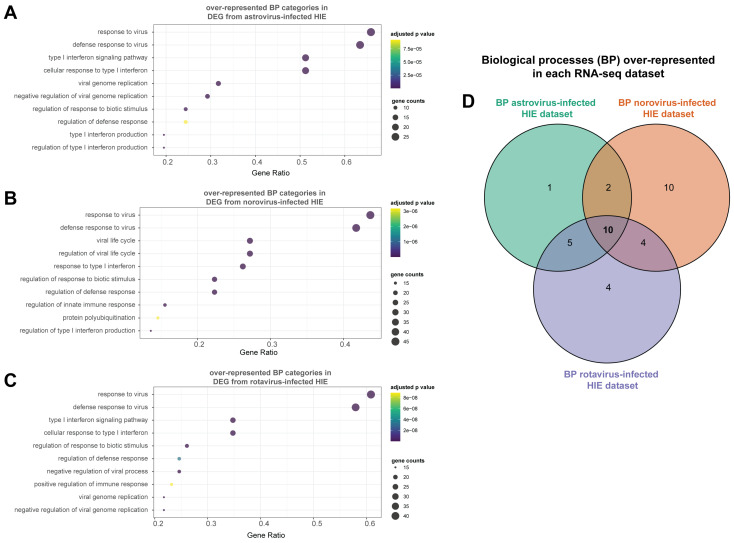
Gene Ontology (GO) over-representation analysis showed that the top over-represented biological processes (BP) for all datasets were associated with defense response to virus and type I IFN responses. Gene ontology (GO) analysis was performed on the list of DEG identified for each independently analyzed dataset to identify over-represented biological processes (BP). The top 10 over-represented BP categories are shown for each dataset as well as the number of genes contributing to the over-representation of each BP. Astrovirus-infected duodenal HIE (**A**), norovirus-infected ileal HIE (**B**), and rotavirus-infected jejunal HIE (**C**) are shown. An adjusted *p*-value cutoff of 0.001 was used to determine significantly over-represented BP. Shared and unique over-represented BP categories across all datasets were determined from the list of over-represented BP categories obtained for each dataset (**D**). Over-represented BP categories were identified with the package clusterProfiler in R.

In addition to specific genes, we also wanted to explore which biological processes were over-represented in the list of DEGs identified in each RNA-seq dataset. Towards that end, we performed a gene ontology (GO) over-representation analysis comparing biological processes (BP). The dot plots highlight the top ten over-represented biological process (BP) categories identified in the list of DEGs for each dataset (Figure 5A–C). 18, 26 and 23 over-represented BP categories were found in astrovirus-infected, norovirus-infected and rotavirus-infected HIEs, respectively, using a FDR cutoff of 0.1% (adjusted *p* value < 0.001) with the Benjamini–Hochberg (BH) step-up procedure (Table 3). Ten over-represented BP categories were shared across all RNA-seq datasets (Figure 5D). The top two over-represented BP categories were the same in all three datasets (“response to virus” [GO:0009615] and “defense response to virus” [GO:0051607]) with a gene ratio between 0.4 and 0.6, showing that close to half of the DEG identified for each dataset fall into these two categories independently of the infecting virus. The next group of over-represented BP categories contained processes associated with a type I IFN response (“regulation of type I interferon production” [GO:0032479] and “type I interferon production” [GO:0032606]) with gene ratios close to 0.2. The total number of over-represented BP categories was similar across the three RNA-seq datasets, even though the number of DEGs identified in the astrovirus-infected duodenal-HIEs was much lower than in the other two datasets (Table 3).

Overall, a shared set of DEGs informing a shared set of biological processes (BP) were identified in the three RNA-seq datasets. This conserved, predominant response of the intestinal epithelium to viral infection is mediated by antiviral defense and IFN signaling pathways.

### 3.5. Distinct DEGs and Biological Processes (BP) were Also Noted for Each Enteric Virus

In addition to shared or partially shared DEGs, we also identified 2, 47, and 18 DEGs uniquely identified in astrovirus-infected, norovirus-infected, and rotavirus-infected HIEs, respectively (Figure 4A, Supplemental Table S3). In the dataset generated from astrovirus-infected duodenal HIEs, two DEGs (*IFI27* and *REV3L*) not overlapping with the other datasets were identified. Interferon Alpha Inducible Protein 27 (IFI27) expression sensitizes cells to apoptotic stimuli [31], which may help the intestinal epithelium to repel astrovirus-infected cells. The dysregulation of REV3L the catalytic subunit of DNA polymerase ζ engages the innate immune response with potential antiviral consequences [32]. However, additional studies are needed to test these hypotheses. A total of 47 DEGs were found exclusively in norovirus-infected ileal HIE, including IFN-induced and -stimulated genes *IFI35* and *IFIT5* as well as the innate immune receptor toll-like receptor (TLR) 3 and several tripartite motif containing (TRIM) genes (*TRIM 14, 21, 25, 34* and *56*). Among the 18 DEGs found exclusively in the rotavirus-infected jejunal HIE dataset were IFN-λ (*IFNL1*, *IFNL2* and *IFNL3*).

**Figure 6:**
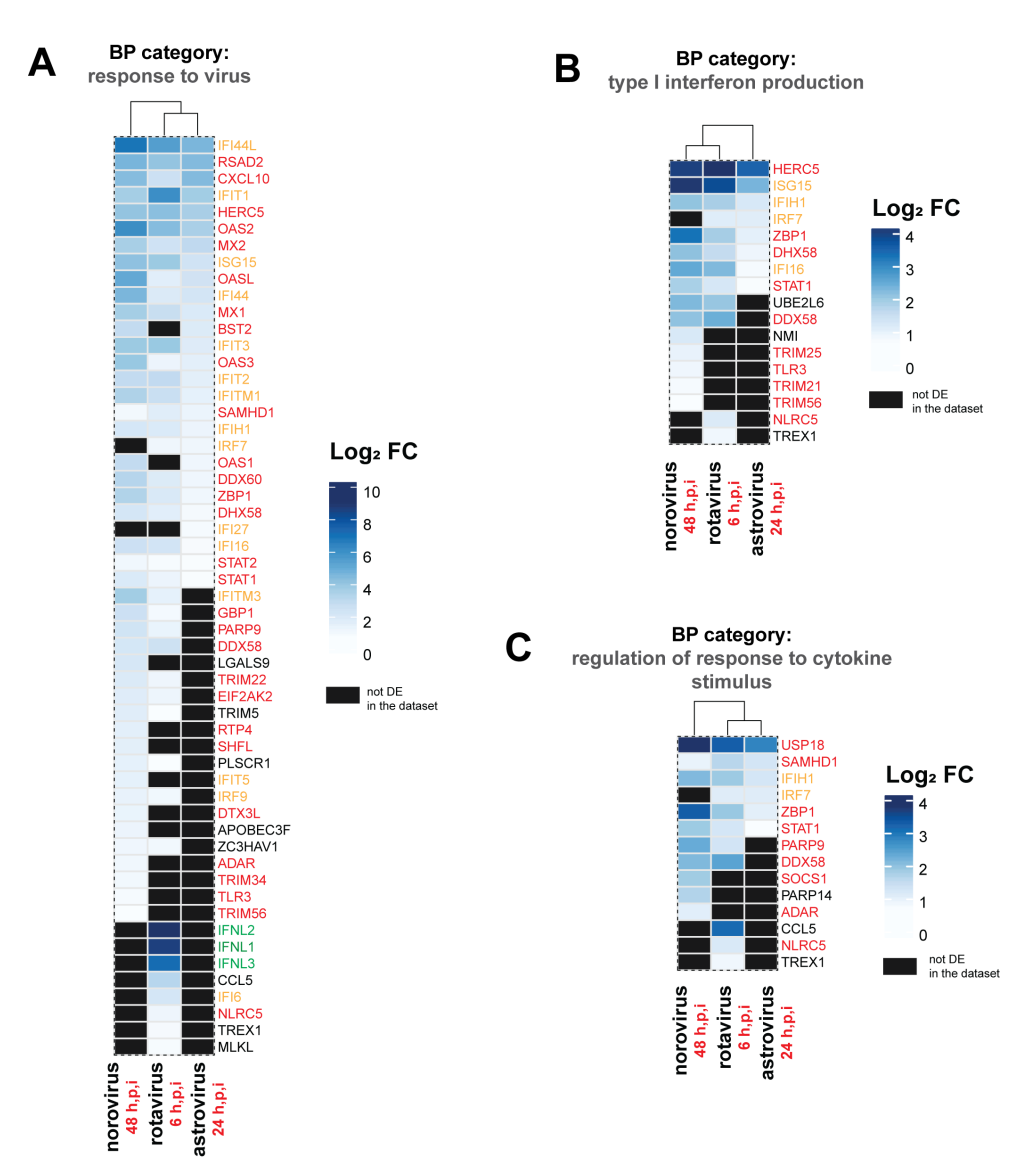
DEG expression patterns in shared over-represented biological processes (BP) in the three RNA-seq datasets after gene ontology (GO) over-representation analysis. Heatmap-like functional classification displays in which differentially expressed genes (DEGs) were assigned to the BP categories “response to virus” (**A**), “type I interferon production” (**B**) and “regulation of response to cytokine stimulus” (**C**). For each dataset, genes that were not differentially expressed for the indicated RNA-seq dataset are indicated in grey. Genes annotated in the Entrez Molecular Sequence Database System as part of IFN responses are highlighted in red. Genes with a symbol containing IFI (IFN-inducible) or ISG (IFN-stimulated genes) are in orange, while genes coding for IFN proteins are in green. Log_2_ fold-change (FC) values within the range of each dataset are shown. Over-represented BP categories were identified with the package clusterProfiler and heatmaps generated with the package ComplexHeatmap in R. The sampling time point in hours post-infection (h.p.i) at which each RNA-seq dataset was generated is indicated.

In line with unique DEGs, there were 1, 4, and 10 over-represented BP that were uniquely identified in each one of the three datasets from astrovirus, rotavirus and norovirus infections, respectively (Figure 5D). However, the majority of these BP categories showed low gene ratios (gene ratio < 0.1). In astrovirus-infected duodenal HIEs “regulation of defense response to virus” [GO:0050688] was the only uniquely over-represented BP category for this dataset (Table 4). However, it was driven by genes not uniquely differentially expressed in this dataset (*IFIT1*, *HERC5*, *DDX60*, *DHX58*, and *STAT1*) (Figure 6). A likely explanation is that the combination of these genes might play a more relevant part of the host response triggered in the intestinal epithelium against astrovirus than norovirus or rotavirus. The chemokine *CCL5* and IFN-λ (*IFNL1-3*) were important for 3 out of the 4 BP over-represented uniquely in rotavirus-infected HIEs (Table 4). These genes were exclusively differentially expressed as part of the HIEs response to rotavirus (Figure 6A). Norovirus infection upregulated the most unique BP categories with 8 out of 10 over-represented BPs including genes encoding TRIM family proteins (Table 4).

While the top shared over-represented BP categories were the same in the three RNA-seq datasets (Figure 5), we noted virus-specific differences in the expression pattern of DEGs assigned to each category (Figure 6). For example, of the 55 genes included in the “response to virus” BP category, 23 were expressed in all datasets, while 13 were differentially expressed in two datasets, and the remaining 19 genes were only differentially expressed in one dataset (Figure 6A). Of note, while the number of DEGs assigned to the BP category “response to virus” in the norovirus-infected HIEs dataset was the largest, the gene ratio was the smallest compared to the other two datasets (Figure 5A–C). This apparent discrepancy is due to the gene ratio being a function of the number of DEGs detected for each dataset. Thus, with the denominator in the norovirus dataset being larger, the ratio ultimately is smaller. A similar pattern of shared, partially shared, and unique DEGs was also observed in other over-represented BPs, including type I IFN production (Figure 6B) and regulation of response to cytokine stimulus (Figure 6C).

In summary, shared and unique DEGs inform similar biological processes that are triggered in HIEs as part of an intestinal epithelial response to viral infection.

### 3.6. Transcription Factor Enrichment Analysis (TFEA) Revealed That the Conserved Intestinal Epithelial Response against the Three Enteric Viruses is Driven by STAT1/STAT2, IRF1, and NFkB

**Figure 7:**
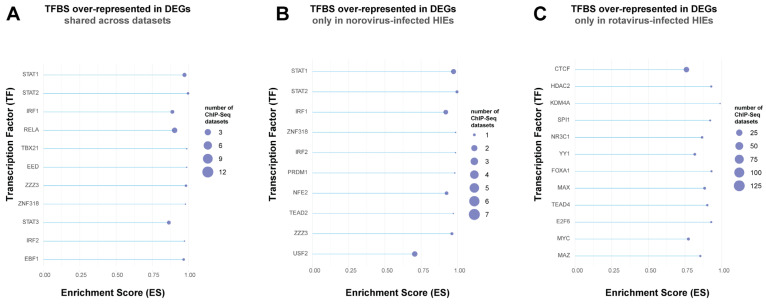
Transcription factor binding site (TFBS) analysis of differentially expressed gene sets (DEGs). Displayed are the enriched TFBS present in the list of 63 DEGs that were shared or partially shared across the three RNA-seq datasets (astrovirus, norovirus and rotavirus-infected HIEs) (**A**), the 47 DEGs only identified in norovirus-infected HIEs (**B**), and the 18 DEGs only identified in rotavirus-infected HIEs (**C**). Enrichment Score (ES) values are displayed for each transcription factor (TF) reflecting the degree to which a TF is significantly enriched (FDR-adjusted *p* value < 0.05) in the subset of DEGs as well as the number of ChIP-seq datasets used in the analysis of the TF. Transcription factor enrichment analysis (TFEA) was performed with the package TFEA.ChIP in R.

After identifying unique and shared DEGs, we next wanted to identify transcription factor binding sites (TFBS) that were enriched in the list of shared and partially shared DEGs across the three RNA-seq datasets. Our rationale was that these DEGs are likely regulated by common transcription factors (TFs) or TFs from the same family to drive conserved responses of HIEs against viral infection. A total of 63 DEGs (shared or partially shared across datasets, Figure 4A) were subjected to Transcription Factor Enrichment Analysis (TFEA). ChIP-seq datasets corresponding to 11 TFs were significantly enriched (FDR-adjusted *p* value < 0.05) in the list of DEGs when compared to non-differentially expressed background genes. Among the top enriched TFBS were ones for STAT1, STAT2, IRF1 and RELA (Figure 7A). Type I IFNs signal through the heterotrimeric factor complex ISGF3 made off phosphorylated STAT1/STAT2 and IRF9 [33]. Thus, the finding of enriched STAT1 and STAT2 TF binding sites is consistent with Type I IFN responses, one of the GO over-represented pathways for all RNA-seq datasets. IRF1 mediates part of HIEs conserved response to these three viruses in the intestinal epithelium. IRF1, a member of the IFN response factor (IRF) family, can activate an antiviral response against a wide range of viruses [34] via the stimulation of cytokines, chemokines and ISGs. It is a potent anti-norovirus effector against human (HNoV) [35] and murine norovirus (MNoV) [36], as well as a target of the rotavirus non-structural protein 1 (NSP1) [37]. A role for IRF1 in controlling astrovirus infection has not been described to date. Furthermore, the NF-κB subunit RELA regulates a predominantly pro-inflammatory set of genes in the course of RIG-I-like receptors (RLR) antiviral response [38]. This includes the target of RELA regulation CXCL11, which was found in the list of DEGs in the astrovirus-infected and norovirus-infected HIEs, further validating the analysis.

In addition, we analyzed the TFBS enriched in the non-shared DEGs, i.e., those identified in a single RNA-seq dataset. This analysis was performed on the 47 and 18 DEGs identified uniquely in norovirus- and rotavirus-infected HIEs, respectively. The number of DEGs identified in the astrovirus-infected HIEs (i.e., 2 DEGs; Figure 4A) was too small for TFEA. We found that TF gene targets identified across ChIP-seq datasets corresponding to 10 and 12 TF (Figure 7B,C) were significantly enriched in the list of unique DEGs identified in norovirus- and rotavirus-infected HIEs, respectively. The top enriched TFBS in the norovirus-infected HIEs dataset were STAT1, STAT2, and IRF1 and largely overlapped with the enriched TFBS in the shared DEGs for all datasets (Figure 7B). Interferon Regulatory Factor 2 (IRF2) was also present and it regulates basal and induced expression of TLR3 in response to IFN stimulation [39]. This is consistent with the finding that *TLR3* was differentially expressed only in norovirus-infected HIEs. Thus, TLR3 activation and signaling appears to be a unique host-response mechanism observed upon norovirus infection in the intestinal epithelium that could be experimentally investigated in the future. Finally, in the list of DEGs identified only in rotavirus-infected HIEs, the top enriched TFBS were CTCF, HDAC2, KDM4A, SPI1, and NR3C1 (Figure 7C). None of these TFBS were enriched in the list of DEGs shared for all viruses or for the norovirus dataset alone, suggesting a unique response of the intestinal epithelium against rotavirus. CCCTC-binding factor (CTCF) is involved in the control of viral gene transcription of several DNA viruses [40] but its potential role during infection by rotavirus, a double-stranded RNA virus, is unknown. This finding might be further explored, especially since there was a strong enrichment of several CTCF ChIP-seq datasets (129 datasets) in the list of DEGs uniquely identified in rotavirus-infected HIEs.

## 4. Discussion

The goal of this study was to identify universal and unique patterns of the intestinal epithelial response to enteric viral infection to contribute to a better understanding of viral pathogenesis. Our meta-analysis draws from studies done at different centers, using HIEs from both different regions in the gut and distinct donors. This biological variability emphasizes the robustness of our ultimate finding, which was that IFNs and ISGs are central to the innate immune response to viral infections within the gut.

Our analysis was aware of the caveats presented from combining distinct RNA-seq datasets: (a) sequenced at different institutions, (b) obtained from HIEs grown from different sections of the small intestine (jejunum, duodenum, and ileum), and (c) collected at different times post-virus infection (6, 24, and 48 hpi with rotavirus, astrovirus and norovirus, respectively). These differences were noticeable when the data was subjected to dimensionality reduction, where t-Distributed Stochastic Neighbor Embedding (t-SNE) showed that the primary factor driving the differences between the three RNA-seq datasets was likely the anatomical origin of the biopsy used to generate HIEs (Figure 1). Despite the variance across datasets due to the biopsy source used to grow HIEs, 89.92% of non-weakly expressed genes were identified in all three datasets (11,262 out of 13,581 genes). This was particularly useful for downstream differential analysis for each one of the RNA-seq datasets, since DEGs were identified from mostly similar lists of genes.

Several over-represented biological processes were part of type I IFN production, signaling, and response (Figure 5) and thus are part of the critical defense response in the intestinal epithelium to enteric viruses. This multigene family encodes 13 partially homologous IFNα subtypes, IFNβ and several other IFN gene products that are less well characterized [31]. However, in this study we were not able to detect either IFNα or IFNβ in any of the RNA-seq datasets. Only IFNε (*IFNE*) was detectable in the datasets generated from astrovirus-infected duodenal HIEs and norovirus-infected ileal HIEs, but *IFNE* was not differentially expressed. This is in contrast to our previously published research, where we found that colonic and ileal HIEs showed detectable IFNβ gene expression in response to astrovirus infection by quantitative PCR [10]. This highlights a caveat of transcriptomic analysis, whereby the findings are influenced by the depth of sequencing required to pick up, in this case, these specific IFN genes. For example, mammalian reovirus infected T84 intestinal cells and enterovirus 71-infected HT29 cells express lower levels of IFNβ transcripts compared to IFNλ [41,42]. As such the level of IFNα and/or IFNβ production in the HIEs could have been below the limit of detection of RNA-sequencing. Similarly, we observed that a type III IFN response was a significant part of the epithelial response to rotavirus, with IFNλ (*IFNL1*, *IFNL2*, and *IFNL3*) being differentially expressed, consistent to what the authors originally reported [12]. Changes in IFNλ however were not subjected to differential expression analysis since these genes had very low relative expression levels and were thus filtered out by our stringent analysis criteria. Thus, a greater sequencing depth would have been required to pick up the IFNλ genes since our research on astroviruses [10] was able to detect IFNλ by quantitative PCR in colonic and ileal HIEs at 24 h post-infection. Nevertheless, it is also possible that the production of IFNλ by HIEs in response to rotavirus might be of a larger magnitude than in response to norovirus or astrovirus infections. Comparative infections with the three viruses in the same HIE line would be required to test this possibility in the future.

Type I and III IFN production and signaling typically occurs in response to stimulation of pattern recognition receptors (PRRs), including toll-like receptors (TLRs). We found TLR3 to be the only TLR differentially expressed and only in ileal HIEs in response to norovirus (≈fold-change of 2). While TLR1, 2 and 3 were detectable across all datasets, they were only differentially expressed in response to norovirus infection. TLR3 is primarily present in the endosomal compartment and senses double-stranded (ds) RNA [42,43]. DsRNA is a RNA virus replication intermediate present during infection with all three viruses. Thus, TLR3 could conceivably function in the antiviral response to all three viruses. However, TLR3 may only be differentially expressed in the norovirus-infected HIEs dataset due to the timing of sample collection (48 h post-infection) since an amplification of the baseline expression level of TLR3 during viral infection of intestinal epithelial cells is part of the ISG response [42]. It is also conceivable that TLR3 might play a more unique role in the epithelial response to norovirus rather than astrovirus and rotavirus. There is precedent from murine norovirus (MNoV) that TLR3 contributes to controlling infection in vivo [44]. Furthermore, a number of ISGs, including IFN-stimulated gene 15 (ISG15), a central player in the host antiviral response [45], and the E3 ubiquitin-protein ligase HERC5, were strongly up-regulated in all datasets and classified as part of the type I IFN production biological process.

In addition to the shared enrichment of DEGs in the type I IFN and response to virus pathways, a variation in the DEGs between the three viruses was also observed. The factors resulting in this variance are likely multi-fold and represent a balance of experimental differences, including in sampling timepoints, and inherent differences in the biology of these three viruses, including their ability to antagonize innate immune responses. For example, the host response to viral infection is dependent on the expression kinetics of type I and III IFNs, among others. The expression of IFNβ and IFNλ mRNA peaked at 16 and 24 hpi, respectively, in intestinal enteroids infected with reovirus [41], consistent with the notion that type III IFN responses are generally of lower magnitude, slower kinetics and longer duration compared to type I IFN responses [46]. Thus, sampling of the IFN responses in the three datasets occurred at different points of the IFN signaling cascade with the astrovirus-infected HIEs being sampled around the peak of IFN expression (i.e., 24 hpi), while rotavirus-infected HIEs were sampled prior (i.e., 6 hpi) and norovirus-infected HIEs sampled after (i.e., 48 hpi) the peak in expression. Thus, the enriched TFBS unique to rotavirus infection could reflect events occurring prior to IFN induction, while the similarities in TFBS enriched during norovirus infection and the shared TFBS are consistent with downstream signaling from the type I and III IFN receptors. Similarly, TLR3 was uniquely identified in the norovirus dataset at 48 hpi, consistent with a previously identified IRF1/type III IFN-mediated positive feedback loop of TLR3 expression that exhibited a delayed peak in TLR3 expression (between 2–3 days pi) relative to IFNλ and IRF1 (1 dpi) in enterovirus EV71-infected intestines [42]. However, these host-driven dynamics are counterbalanced by virus-encoded proteins since pathogenic viruses have evolved multiple strategies to overcome innate host defenses and establish successful infection. In case of rotavirus, several strategies to disrupt host defense responses have been identified. These include degradation of the mitochondrial antiviral signaling protein (MAVS) by the RNA capping enzyme VP3 [47] to limit viral sensing, degradation of type I, II, III IFN receptors [48], and nonstructural protein NSP1-mediated degradation of IRF3, IRF5, and IRF7 [37,49] or inhibition of NFkB signaling via degradation of the β-transducin repeat-containing protein (β-TrCP) [50,51,52]. Much less is known about human norovirus antagonism. A recent study did not observe differences in STAT1-deficient vs wild-type HIE infected with the same genotype used in the RNA-seq study (GII.4), while a different genotype (GII.3) was sensitive, suggesting GII.4 HNoV limits IFN signaling [28]. While the human norovirus p48 (NS1/2) and p22 (NS4) proteins interfere with intracellular protein trafficking when overexpressed in cells [53], whether they block IFN secretion during infection remains to be explored. Whether human astroviruses employ mechanisms to antagonize IFN responses remains unknown. Thus, the interplay between viral and host factors likely drives differences in differential expression between the various virus-infected HIEs datasets.

Overall, while a comparative analysis of dissimilar RNA-seq datasets from the literature has limitations, our processing pipeline and filters (removal of weakly-expressed genes, higher FDR cutoffs) limited these caveats and allowed us to draw several conclusions. Specifically, we identified key shared players across all datasets, which are part of the response to virus (e.g., IF44, IF44L, STAT1, STAT2, MX1, and MX2), demonstrating that HIEs employ a generic antiviral response. However, this is augmented by more tailored responses depending on the virus (e.g., IFI27 in response to astrovirus, and TLR3 in response to norovirus). We hope this comparative study provides a useful resource to the scientific community that stimulates further explorations into common and unique aspects of the host response to virus infections in the human intestinal epithelium. These studies should be complemented by comparative studies between different viral pathogens that cause gastroenteritis in the same host genetic background.

## Figures and Tables

**Table 1:** Differential expression analysis summary. Differential analysis of RNA-seq data was performed on the three published RNA-seq datasets. Listed are the total number non-weakly expressed genes that were analyzed in each dataset to determine differentially expressed genes (DEG).

Differential Expression Analysis Summary					
Dataset	HIEs	# Genes Analyzed	DEGs	Up-Regulated-DEGs	Down-Regulated DEGs
astrovirus-infected	duodenal	11,824	42	42	0
norovirus-infected	ileal	12,917	109	109	0
rotavirus-infected	jejunal	12,416	75	71	4

**Table 2:** Differentially expressed genes (DEGs) shared in all datasets that are linked to IFN responses. This list contains DEGs that were identified in each dataset and thus thought to contribute to the conserved antiviral response of human intestinal enteroids (HIEs) as well as their association with Type I, II and III IFN responses according to the Interferome database.

Shared Differentially Expressed Genes (DEGs) and IFN Responses						
HGNC Symbol	Ensemble Gene ID	Entrez Gene ID	Entrez Gene Description	Type I IFN	Type II IFN	Type III IFN
**BATF2**	ENSG00000168062	116071	basic leucine zipper ATF-like transcription factor 2	yes	yes	yes
**CMPK2**	ENSG00000134326	129607	cytidine/uridine monophosphate kinase 2	yes	yes	no
**CXCL10**	ENSG00000169245	3627	C-X-C motif chemokine ligand 10	yes	yes	no
**DDX58**	ENSG00000107201	23586	DExD/H-box helicase 58	yes	yes	yes
**DDX60**	ENSG00000137628	55601	DExD/H-box helicase 60	yes	yes	no
**DHX58**	ENSG00000108771	79132	DExH-box helicase 58	yes	yes	no
**EPSTI1**	ENSG00000133106	94240	epithelial stromal interaction 1	yes	yes	yes
**HELZ2**	ENSG00000130589	85441	helicase with zinc finger 2	yes	yes	yes
**HERC5**	ENSG00000138646	51191	HECT and RLD domain containing E3 ubiquitin protein ligase 5	yes	yes	no
**HSH2D**	ENSG00000196684	84941	hematopoietic SH2 domain containing	yes	yes	no
**IFI16**	ENSG00000163565	3428	interferon gamma inducible protein 16	yes	yes	no
**IFIH1**	ENSG00000115267	64135	interferon induced with helicase C domain 1	yes	yes	yes
**IFIT1**	ENSG00000185745	3434	interferon induced protein with tetratricopeptide repeats 1	yes	yes	yes
**IFIT2**	ENSG00000119922	3433	interferon induced protein with tetratricopeptide repeats 2	yes	yes	yes
**IFIT3**	ENSG00000119917	3437	interferon induced protein with tetratricopeptide repeats 3	yes	yes	yes
**IFITM1**	ENSG00000185885	8519	interferon induced transmembrane protein 1	yes	yes	no
**ISG15**	ENSG00000187608	9636	ISG15 ubiquitin like modifier	yes	yes	yes
**LAMP3**	ENSG00000078081	27074	lysosomal associated membrane protein 3	yes	yes	no
**LAP3**	ENSG00000002549	51056	leucine aminopeptidase 3	yes	yes	no
**MX1**	ENSG00000157601	4599	MX dynamin like GTPase 1	yes	yes	yes
**MX2**	ENSG00000183486	4600	MX dynamin like GTPase 2	yes	yes	no
**OAS2**	ENSG00000111335	4939	2′-5′-oligoadenylate synthetase 2	yes	yes	no
**OAS3**	ENSG00000111331	4940	2′-5′-oligoadenylate synthetase 3	yes	yes	yes
**OASL**	ENSG00000135114	8638	2′-5′-oligoadenylate synthetase like	yes	yes	yes
**PARP10**	ENSG00000178685	84875	poly(ADP-ribose) polymerase family member 10	yes	yes	yes
**RSAD2**	ENSG00000134321	91543	radical S-adenosyl methionine domain containing 2	yes	yes	no
**SAMHD1**	ENSG00000101347	25939	SAM and HD domain containing deoxynucleoside triphosphate triphosphohydrolase 1	yes	yes	no
**STAT1**	ENSG00000115415	6772	signal transducer and activator of transcription 1	yes	yes	yes
**STAT2**	ENSG00000170581	6773	signal transducer and activator of transcription 2	yes	yes	yes
**USP18**	ENSG00000184979	11274	ubiquitin specific peptidase 18	yes	yes	yes
**XAF1**	ENSG00000132530	54739	XIAP associated factor 1	yes	yes	no
**ZBP1**	ENSG00000124256	81030	Z-DNA binding protein 1	yes	yes	no

**Table 3:** Summary of Gene Ontology (GO) over-representation analysis. Over-representation analysis of biological processes (BP) was performed with the package ClusterProfiler in R on three published datasets of virus infected HIEs. An FDR cutoff of 0.1% (adjusted *p* value < 0.001) with the Benjamini–Hochberg (BH) step-up procedure was used to determine significantly over-represented BP. Listed are the total number of DEGs identified for each dataset as well as the number of DEGs that were assigned to a BP after over-representation analysis. For each dataset, the number of BP over-represented are also shown.

Gene Ontology (GO) Over-Representation Analysis Summary				
Dataset	HIE	# DEGs	# DEGs Assigned to BP Categories	# BP Categories Over-Represented
astrovirus-infected	duodenal	42	41	18
norovirus-infected	ileal	109	103	26
rotavirus-infected	jejunal	75	69	23

**Table 4:** Over-representation analysis of biological processes (BP) with the package ClusterProfiler in R was used to identify uniquely over-represented BPs for each dataset. A false discovery rate (FDR) adjusted *p*-value cutoff of <0.001 using the Benjamini–Hochberg (BH) step-up procedure was used to determine significant over-represented BP categories. BP categories unique to each dataset are indicated including the gene ontology (GO) term ID and description as well as the genes assigned to the GO term ID.

Uniquely Over-Represented Biological Processes (BPs) for each Dataset Analyzed							
Dataset	HIEs	GO Term ID	GO Term Description	Gene Ratio	Gene Count	adj. *p*-Value	Gene ID
astrovirus-infected	duodenal	GO:0050688	regulation of defense response to virus	0.12	5	0.00012	*IFIT1,HERC5,DDX60,DHX58,STAT1*
norovirus-infected	ileal	GO:1903900	regulation of viral life cycle	0.27	28	0.00000	*OAS2,OASL,RSAD2,ISG15,LAMP3,OAS3,IFITM3,IFIT1,MX1,* *IFITM1,BST2,OAS1,IFI16,LGALS9,TRIM22,PARP10,EIF2AK2,* *TRIM5,SHFL,PLSCR1,IFIT5,APOBEC3F,ZC3HAV1,ADAR,* *TRIM34,TRIM25,TRIM14,TRIM21*
norovirus-infected	ileal	GO:0019058	viral life cycle	0.27	28	0.00000	*OAS2,OASL,RSAD2,ISG15,LAMP3,OAS3,IFITM3,IFIT1,MX1,* *IFITM1,BST2,OAS1,IFI16,LGALS9,TRIM22,PARP10,EIF2AK2,* *TRIM5,SHFL,PLSCR1,IFIT5,APOBEC3F,ZC3HAV1,ADAR,* *TRIM34,TRIM25,TRIM14,TRIM21*
norovirus-infected	ileal	GO:0034340	response to type I interferon	0.26	27	0.00000	*OAS2,OASL,RSAD2,ISG15,OAS3,USP18,IFIT3,IFITM3,XAF1,* *IFIT1,MX1,MX2,IFITM1,ZBP1,BST2,IFIT2,OAS1,STAT1,IFI35,* *SHFL,IRF9,SP100,ADAR,STAT2,SAMHD1,TRIM56,PSMB8*
norovirus-infected	ileal	GO:0000209	protein polyubiquitination	0.15	15	0.00000	*HERC5,UBE2L6,HERC6,TRIM22,PARP10,TRIM5,DTX3L,NMI,* *RNF213,TRIM34,PSMB9,TRIM14,TRIM21,TRIM56,PSMB8*
norovirus-infected	ileal	GO:0002683	negative regulation of immune system process	0.12	12	0.00020	*CD274,BST2,GBP1,IFI16,DHX58,LGALS9,SOCS1,PARP14,NMI,* *ADAR,SAMHD1,TLR3*
norovirus-infected	ileal	GO:0051701	interaction with host	0.11	11	0.00002	*IFITM3,IFIT1,IFITM1,LGALS9,TRIM22,EIF2AK2,TRIM5,TRIM34,* *TRIM25,TRIM14,TRIM21*
norovirus-infected	ileal	GO:0043123	positive regulation of I-kappaB kinase/NF-kappaB signaling	0.10	10	0.00006	*BST2,LGALS9,TRIM22,TRIM5,IFIT5,TRIM34,TRIM25,TLR3,* *TRIM14,TRIM21*
norovirus-infected	ileal	GO:0046718	viral entry into host cell	0.09	9	0.00001	*IFITM3,IFITM1,LGALS9,TRIM22,TRIM5,TRIM34,TRIM25,* *TRIM14,TRIM21*
norovirus-infected	ileal	GO:0051092	positive regulation of NF-kappaB transcription factor activity	0.09	9	0.00007	*LGALS9,TRIM22,EIF2AK2,TRIM5,TRIM34,TRIM25,TLR3,* *TRIM14,TRIM21*
norovirus-infected	ileal	GO:0071360	cellular response to exogenous dsRNA	0.04	4	0.00029	*IFIT1,DDX58,IFIH1,TLR3*
rotavirus-infected	jejunal	GO:0048525	negative regulation of viral process	0.25	17	0.00000	*IFNL3,IFIT1,OAS2,RSAD2,ISG15,CCL5,MX1,IFITM1,IFI16,* *OASL,IFITM3,OAS3,STAT1,EIF2AK2,ZC3HAV1,TRIM5,PLSCR1*
rotavirus-infected	jejunal	GO:0050778	positive regulation of immune response	0.23	16	0.00000	*IFNL2,IFNL1,IFNL3,RSAD2,CCL5,DDX58,IFI16,CD274,ZBP1,* *PARP9,NLRC5,IRF7,GBP1,TREX1,TRIM5,PLSCR1*
rotavirus-infected	jejunal	GO:0007259	receptor signaling pathway via JAK-STAT	0.10	7	0.00002	*IFNL2,IFNL1,IFNL3,CCL5,PARP9,STAT1,STAT2*
rotavirus-infected	jejunal	GO:0002753	cytoplasmic pattern recognition receptor signaling pathway	0.07	5	0.00110	*DDX58,IFIH1,DDX60,DHX58,IRF7*

## Data Availability

The datasets that have been used in this study are publicly available from the GEO and EMBL-EBI. The source code used for analysis of the datasets and to generate the figures in this study are available at: https://gitlab.com/rjcieza/HIEs-GI-viruses-comparative-transcriptomic.git accessed on 30 March 2021.

## Supplementary Materials

The following are available online at https://www.mdpi.com/article/10.3390/v13061059/s1, Figure S1: Samples across the three datasets showed divergence due to tissue origin of the HIE line and due to viral infection when looking at each dataset individually, Table S1: Sequence read archive (SRA) list of all samples analyzed in this study, Table S2: Non-weakly expressed genes shared in all RNA-seq datasets and partially shared across datasets, Table S3: Uniquely differentially expressed genes (DEGs) for each RNA-seq dataset analyzed.
