# Supplementary figures and images for: Comparative Analysis of Public RNA-Sequencing Data from Human Intestinal Enteroid (HIEs) Infected with Enteric RNA Viruses Identifies Universal and Virus-Specific Epithelial Responses
Source: Viruses. 2021 Jun 3;13(6):1059. doi: 10.3390/v13061059 (PMC8227290; doi:10.3390/v13061059)

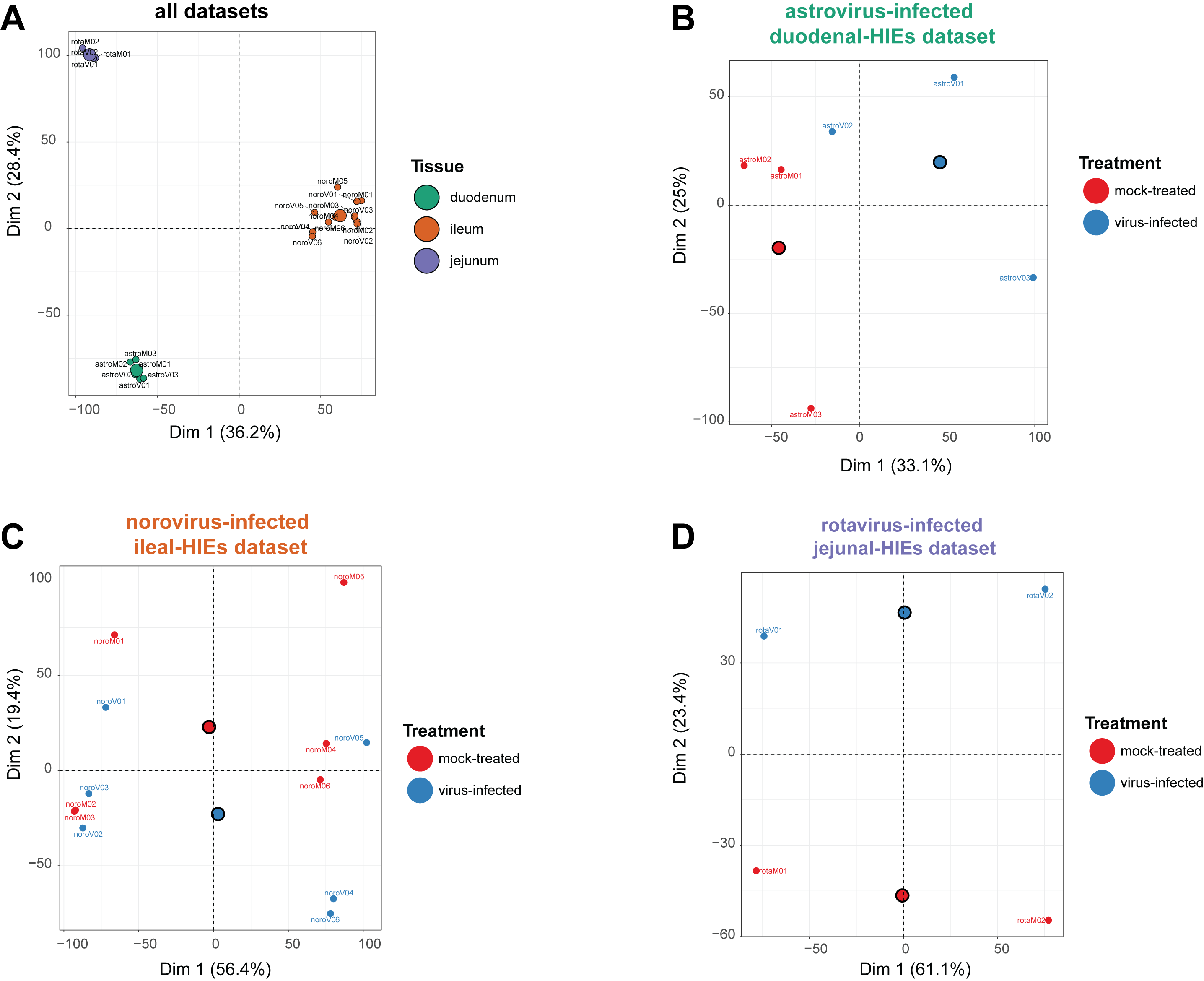

Supplement: Supplementary file 1 [file viruses-13-01059-s001.zip › supplemental_figure_01.png]
